# Supplementary figures and images for: Evaluating poverty alleviation strategies in a developing country
Source: PLoS One. 2020 Jan 13;15(1):e0227176. doi: 10.1371/journal.pone.0227176 (PMC6957162; doi:10.1371/journal.pone.0227176)

S1 Fig. A simple FCM obtained during the workshop conducted in phase 1

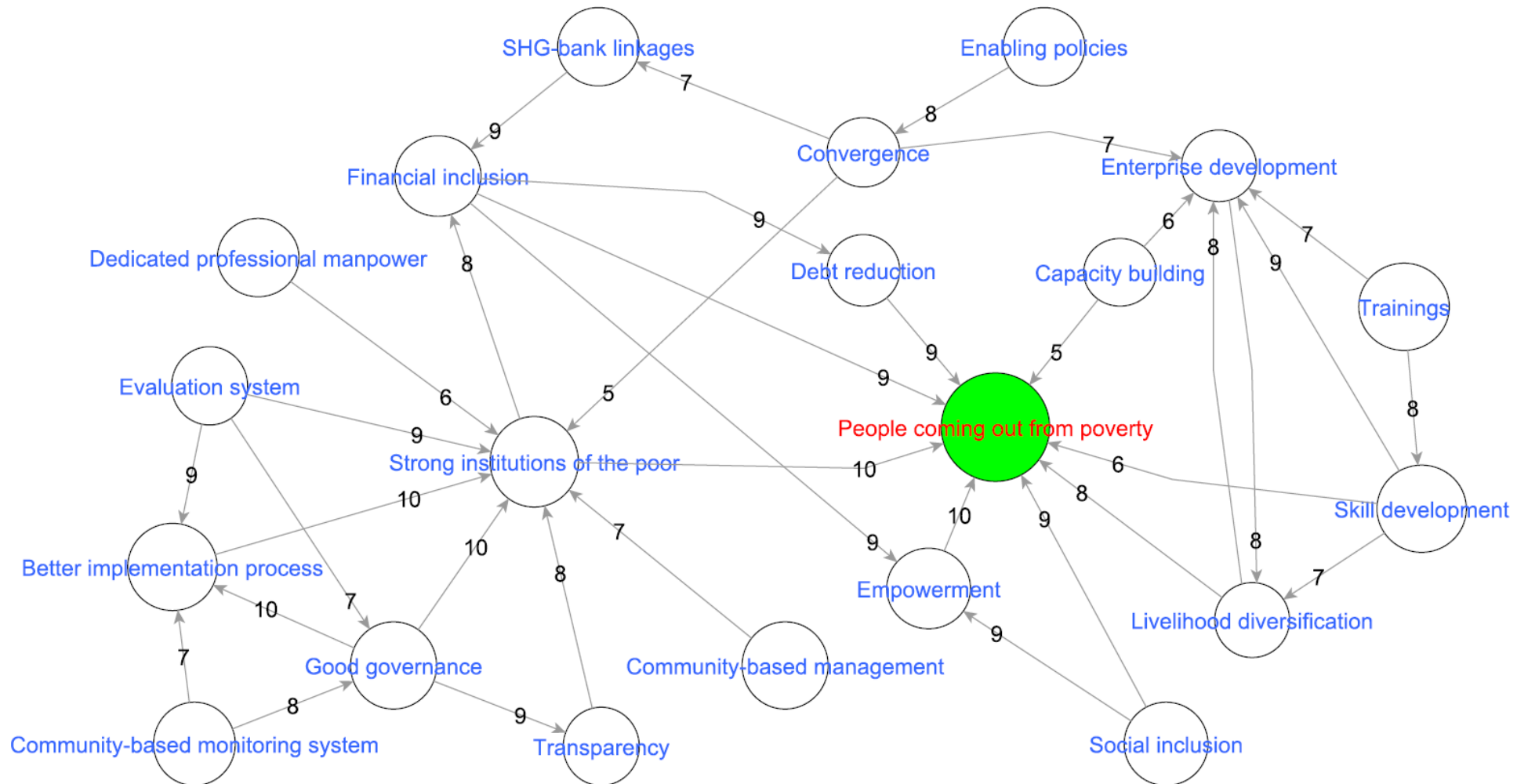

Supplement: S1 Fig — (PDF) [file pone.0227176.s001.pdf]
