# Supplementary material for: Evaluating poverty alleviation strategies in a developing country
Source: PLoS One. 2020 Jan 13;15(1):e0227176. doi: 10.1371/journal.pone.0227176 (PMC6957162; doi:10.1371/journal.pone.0227176)
Supplement: S2 Fig — (PDF) [file pone.0227176.s002.pdf]

S2 Fig. The survey instrument used during the stage 2 of data collection

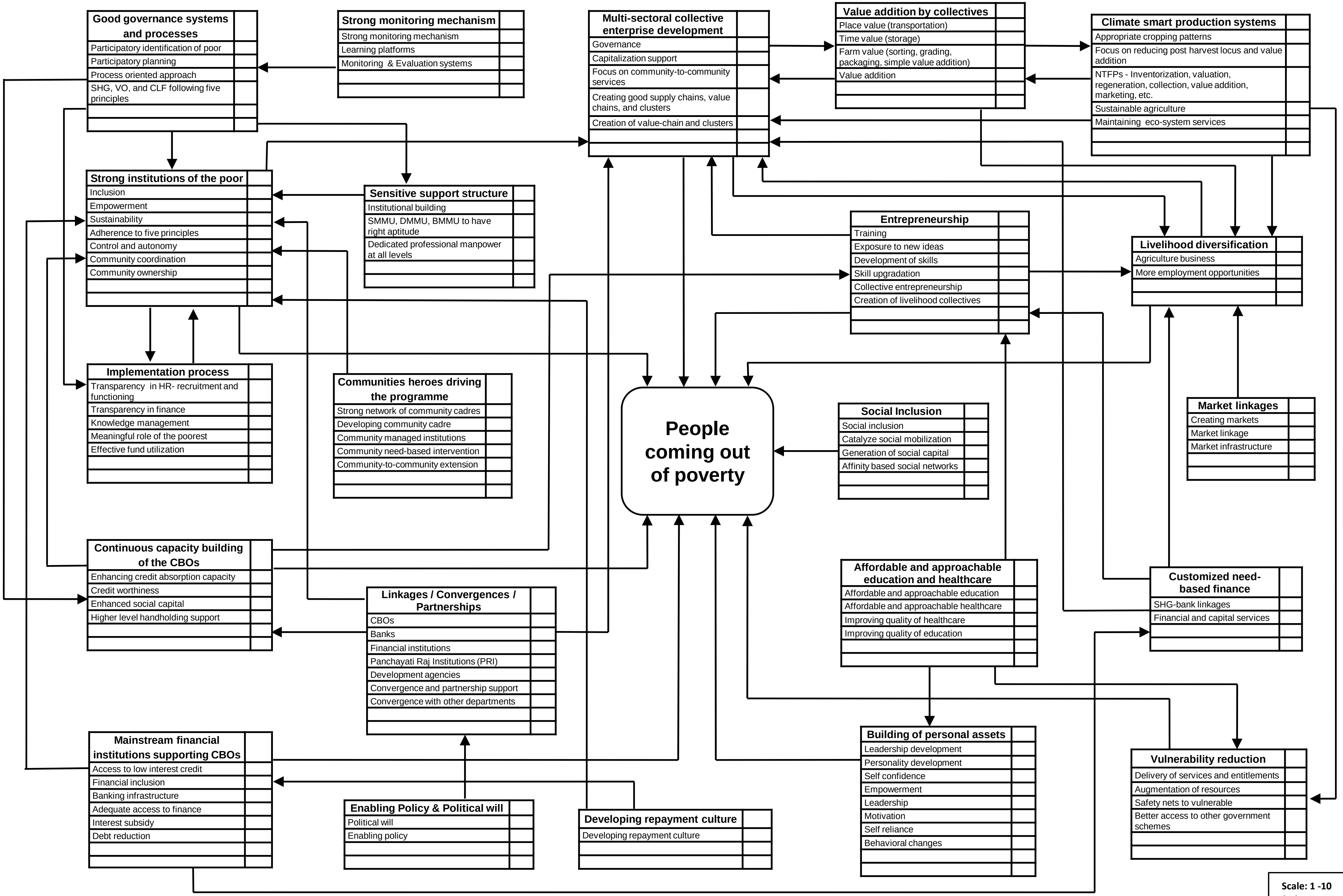

Scale: 1 -10
1= Lowest
10 = Highest

Name of Respondent:
Respondent's Mobile No.:
Gender: Male / Female
State:
District:
Organization' s Name:
Designation:
NRLM Experience (Months):
