# Supplementary material for: Evaluating poverty alleviation strategies in a developing country
Source: PLoS One. 2020 Jan 13;15(1):e0227176. doi: 10.1371/journal.pone.0227176 (PMC6957162; doi:10.1371/journal.pone.0227176)
Supplement: S3 Fig — (PDF) [file pone.0227176.s003.pdf]

S3 Fig. A list of all the concept categories and sub-concepts after data collection

CC: People coming out of poverty

C5: Good governance systems and processes
- Participatory identification of poor
- Participatory planning
- Process oriented approach
- SHG, VO, and CLF following five principles
- VO and CLF following seven principles
- Maashik pratividan regularity
- Grading of CBOs
- Rotation of leaders

C2: Strong institutions of the poor
- Inclusion
- Empowerment
- Sustainability
- Adherence to five principles
- Control and Autonomy
- Special case to vulnerable and marginal household
- Community coordination
- Community ownership
- Supporting system and knowledge that aids systems transparency
- Participatory decision making
- Cohesiveness

C7: Implementation process
- Transparency in HR- recruitment and functioning
- Transparency in finance
- Knowledge management
- Meaningful role of the poorest
- Effective fund utilization
- Focus on process to recruit HR
- Community-driven implementation process
- Community-induced implementation process
- Transparency in procurement process

C3: Communities heroes driving the programme
- Strong network of community cadres
- Developing community cadre
- Community managed institutions
- Community need based intervention
- Community to community extension

C10: Mainstream financial institutions supporting CBOs
- Access to low interest credit
- Financial inclusion
- Banking infrastructure
- Adequate access to finance
- Interest subsidy
- Debt reduction
- Lawyer understanding at bank level
- Higher presence of commercial banks in rural areas
- Business correspondent
- Timely availability of finance

C6: Strong monitoring mechanism
- Strong monitoring mechanism
- Learning platforms
- Monitoring and evaluation systems
- Understanding about ways to enhance efficiency
- Effective MIS – SHG to NMMUs
- Community-based monitoring system

C1: Sensitive support structure
- Institutional building
- SMMU, DMMU, BMMU to have right aptitude
- Dedicated professional manpower at all levels
- Long-term aspirations of people for association with institution working on poverty reduction
- Better remuneration to attract talent for rural areas
- Interactive platform with district administration/ PRI members

C4: Continuous capacity building of the CBOs
- Enhancing credit absorption capacity
- Credit worthiness
- Enhanced social capital
- Higher level handholding support
- Adequate knowledge base that supports capacity building
- Dedicated team to work on modules
- Digitized platform for CBOs

C8: Linkages/ Convergences/ Partnerships
- CBOs
- Banks
- Financial institutions
- Panchayati Raj Institutions (PRI)
- Development agencies
- Convergence and partnership support
- Convergence with other departments
- Art of working with community institutions needs to imparted to other depts. as well

C9: Enabling policy and political will
- Political will
- Enabling policy
- Timely and adequate resource allocation by govt. institutions
- Fewer political influence

C15: Multi-sectoral collective enterprise development
- Governance
- Capitalization support
- Focus on community-to-community services
- Creating good supply chains, value chains, and clusters
- Creation of value-chain and clusters
- Up-to-date warehousing

C12: Developing repayment culture
- Developing repayment culture
- Need to be sensitive to situational defaulters
- Being responsible for repayment
- Trust building among the members for repayment

C22: Adequate knowledge base
- Academic understanding
- Analysis of resources required to facilitate social and economic inclusion of poor
- Investment in facilitating knowledge base
- Prepared use for social infrastructure to facilitate social prosperity

C16: Value addition by collectives
- Place value (transportation)
- Time value (storage)
- Farm value (sorting, grading, packaging, simple value addition)
- Value addition
- Collective purchase

C14: Entrepreneurship
- Training
- Exposure to new ideas
- Development of skills
- Skill upgradation
- Collective entrepreneurship
- Creation of livelihood collectives
- Incubating and promoting innovations suited for rural areas
- Incubation fund/ start-up fund
- Counseling centers
- Community mobilization drives

C19: Social inclusion
- Social inclusion
- Catalyze social mobilization
- Generation of social capital
- Affinity based social networks
- Social infrastructure
- Inclusion of vulnerable section (old age, handicapped, etc.)

C20: Affordable and approachable education and healthcare
- Affordable and approachable education
- Affordable and approachable healthcare
- Improving quality of healthcare
- Improving quality of education
- Integration of health and nutrition services
- Link health and nutrition services with livelihoods

C21: Building of personal assets
- Leadership development
- Personality development
- Self confidence
- Empowerment
- Motivation
- Self-reliance
- Behavioral changes
- Companionship

C18: Climate smart production systems
- Appropriate cropping patterns
- Focus on reducing post-harvest locus and value addition
- NTFPs - Inventorization, valuation, regeneration, collection, value addition, marketing, etc.
- Sustainable agriculture
- Maintaining eco-system services
- Better livestock management
- Eco-friendly activities

C13: Livelihood diversification
- Agriculture business
- More employment opportunities
- Micro-enterprise development
- Better premium to agricultural products
- Integrated livelihood planning
- Non-farm enterprise
- Developing basket of livelihoods

C17: Market linkages
- Creating markets
- Market linkage
- Market infrastructure
- Backward linkages
- Formation of village haats
- Market knowledge

C11: Customized need-based finance
- SHG-Bank linkages
- Financial and capital services
- Adequate investment by govt. in community institutions
- Enterprise-led finance
- Planning and sectoral based finance

C23: Vulnerability reduction
- Delivery of services and entitlements
- Augmentation of resources
- Safety nets to vulnerable
- Better access to other government schemes
- Understanding about what it takes to deliver services and entitlements
- Disaster management
