# Supplementary material for: Evaluating poverty alleviation strategies in a developing country
Source: PLoS One. 2020 Jan 13;15(1):e0227176. doi: 10.1371/journal.pone.0227176 (PMC6957162; doi:10.1371/journal.pone.0227176)

S4 Fig. Sensitivity analysis of the system

Activation value (0.1): Modified activation rule with sigmoid transformation function

Values of concepts

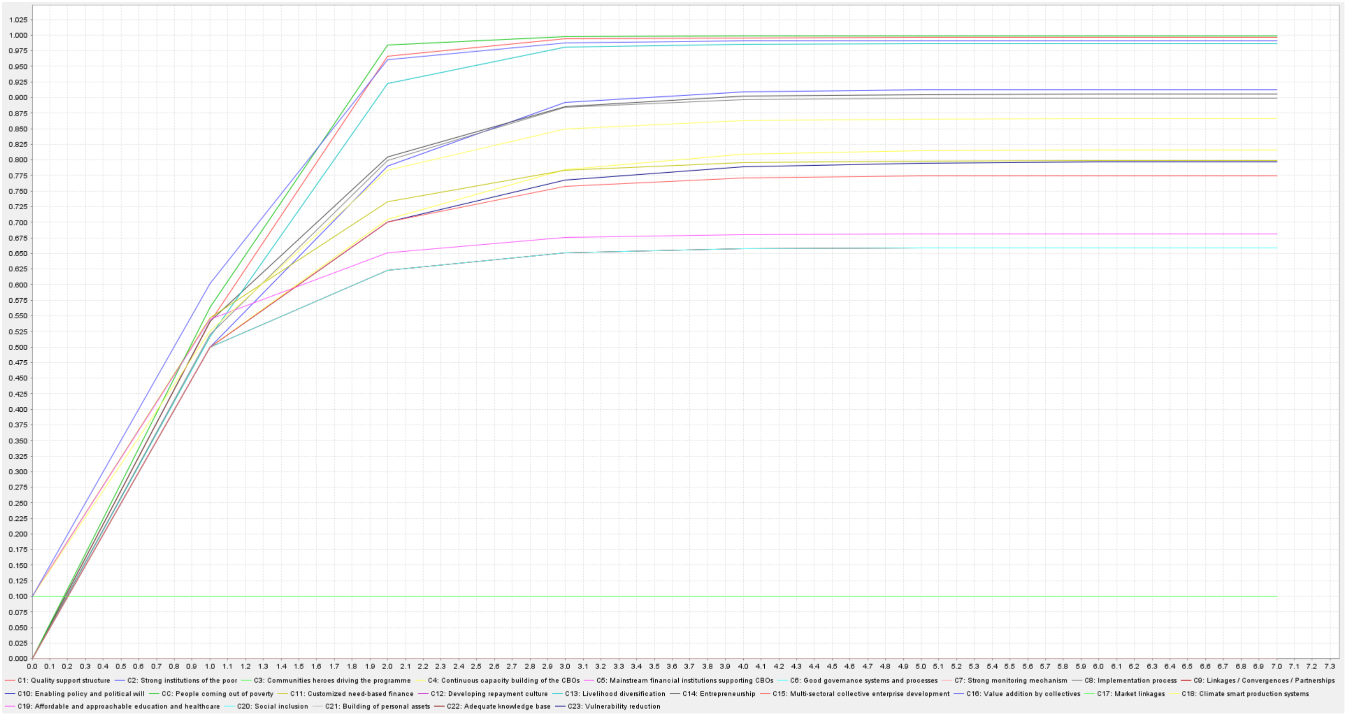

Number of iterations

Activation value (0.2): Modified activation rule with sigmoid transformation function

Values of concepts

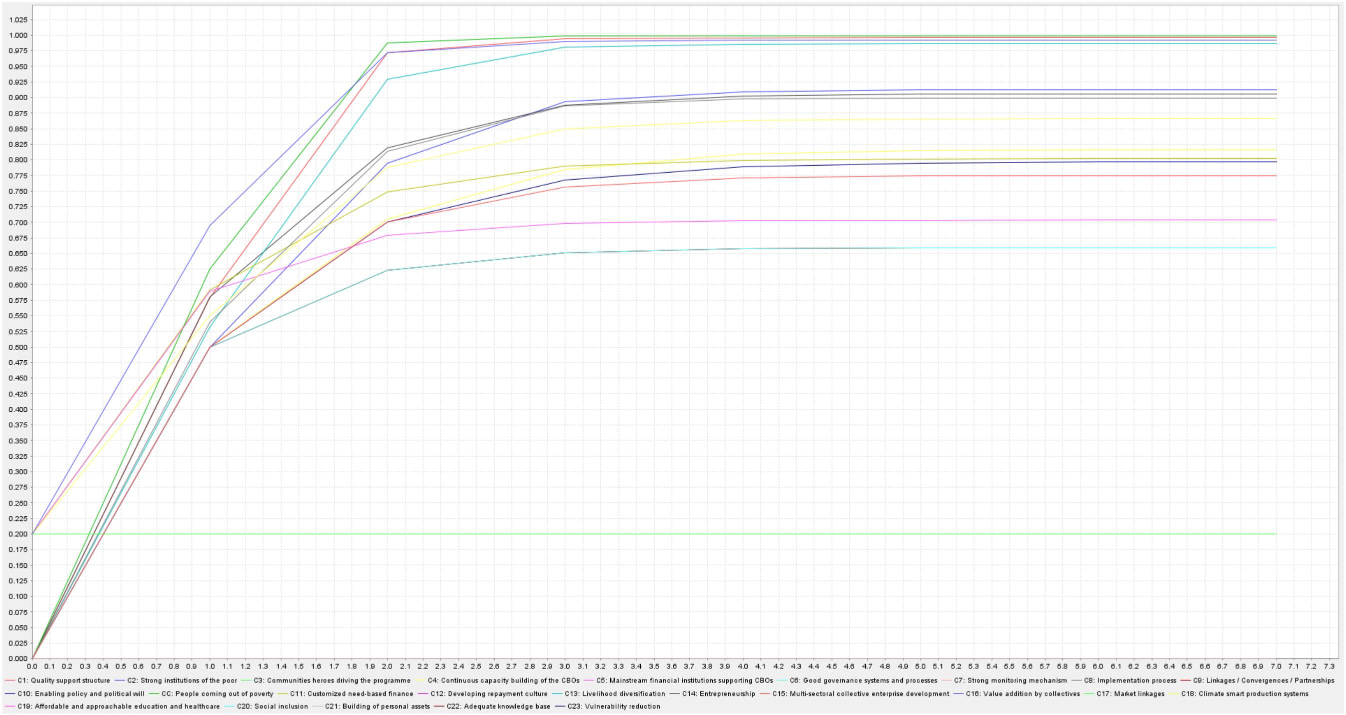

Number of iterations

Activation value (0.3): Modified activation rule with sigmoid transformation function

Values of concepts

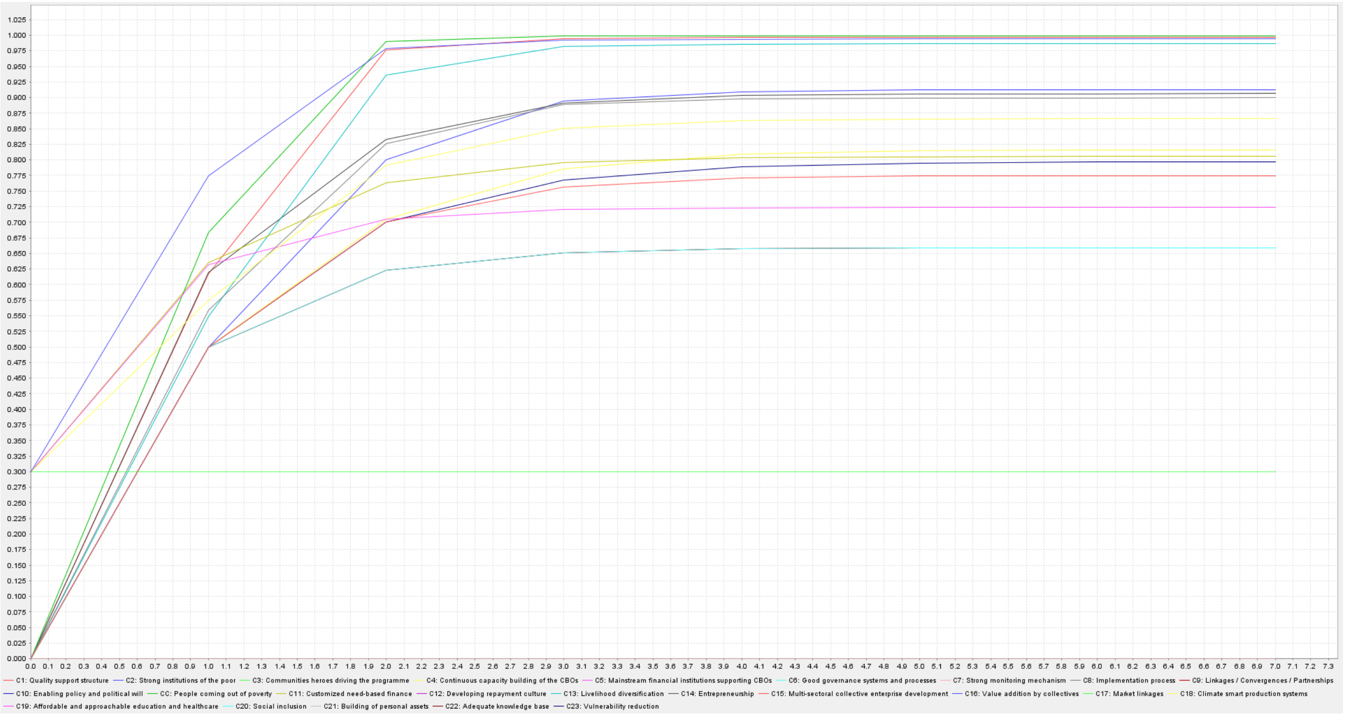

Number of iterations

Activation value (0.4): Modified activation rule with sigmoid transformation function

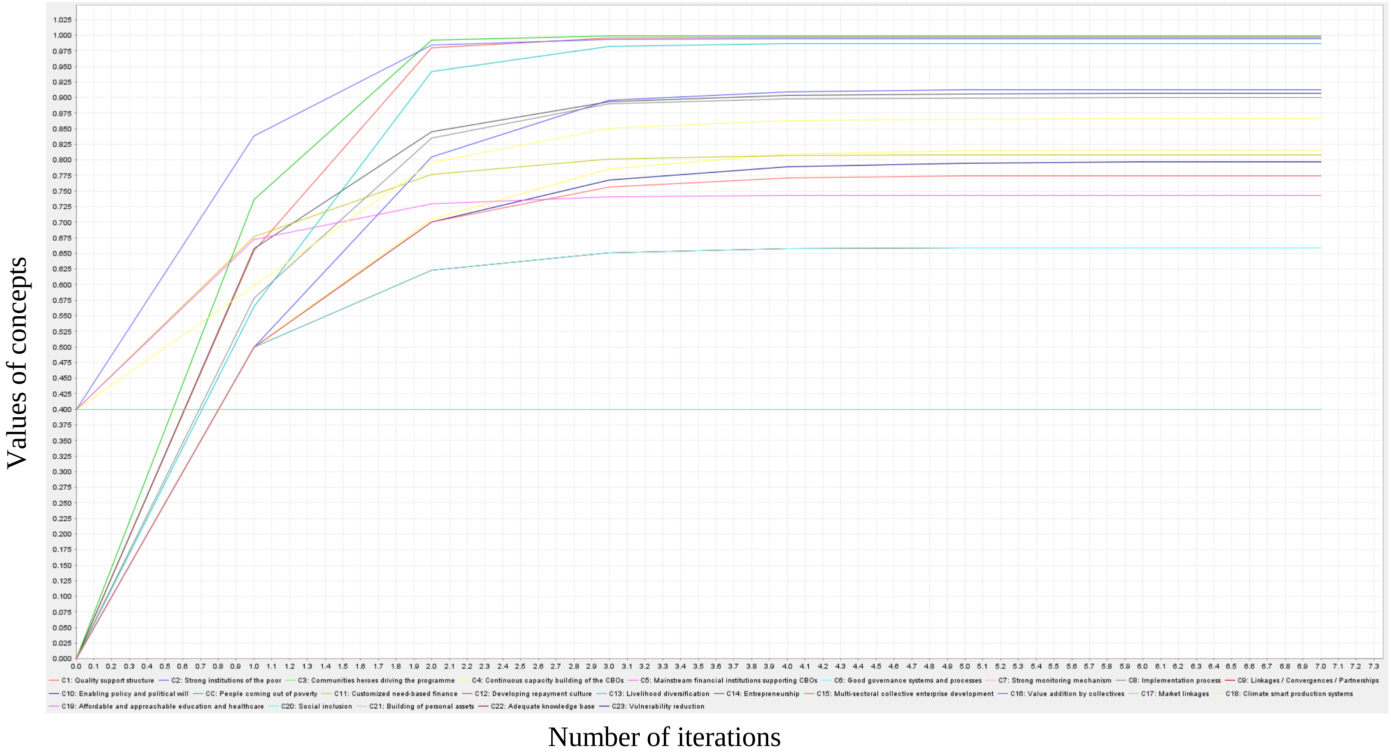

Activation value (0.5): Modified activation rule with sigmoid transformation function

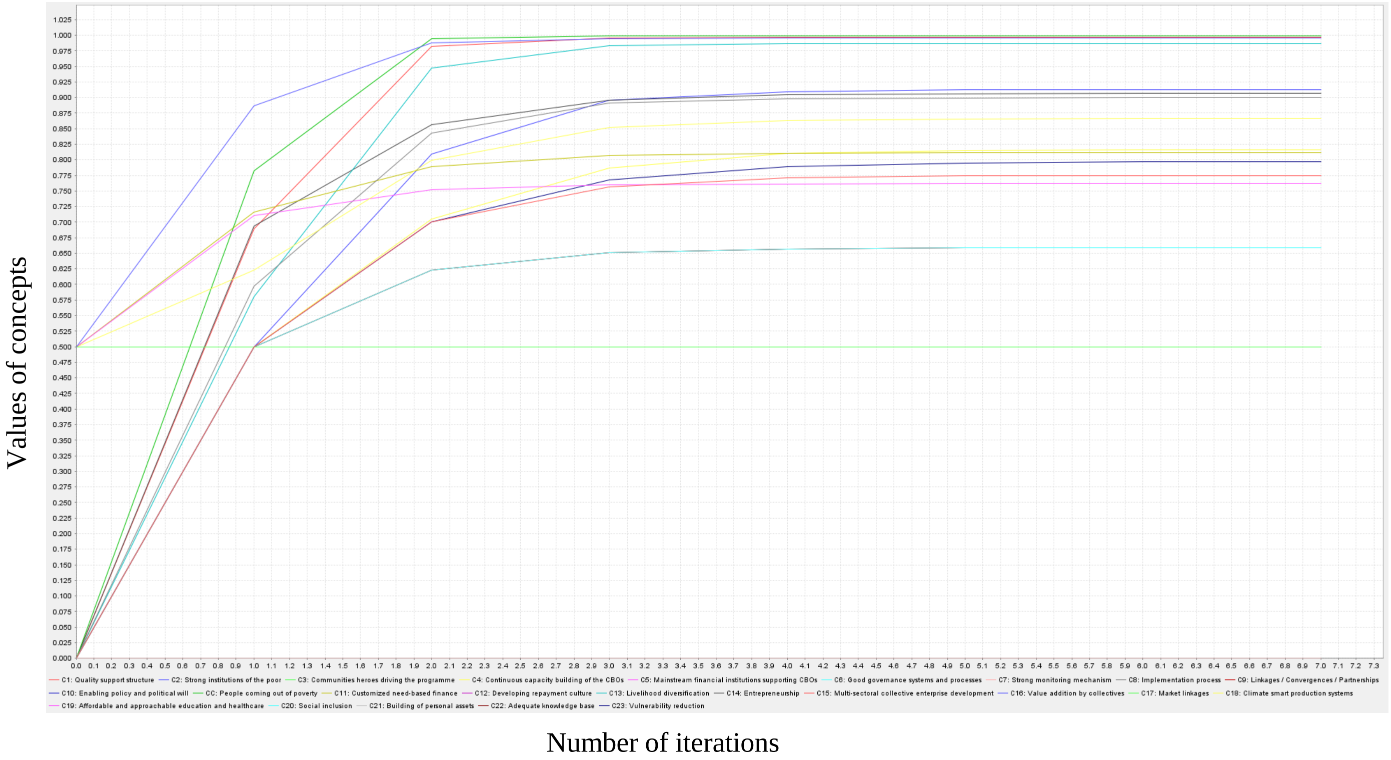

Activation value (0.6): Modified activation rule with sigmoid transformation function

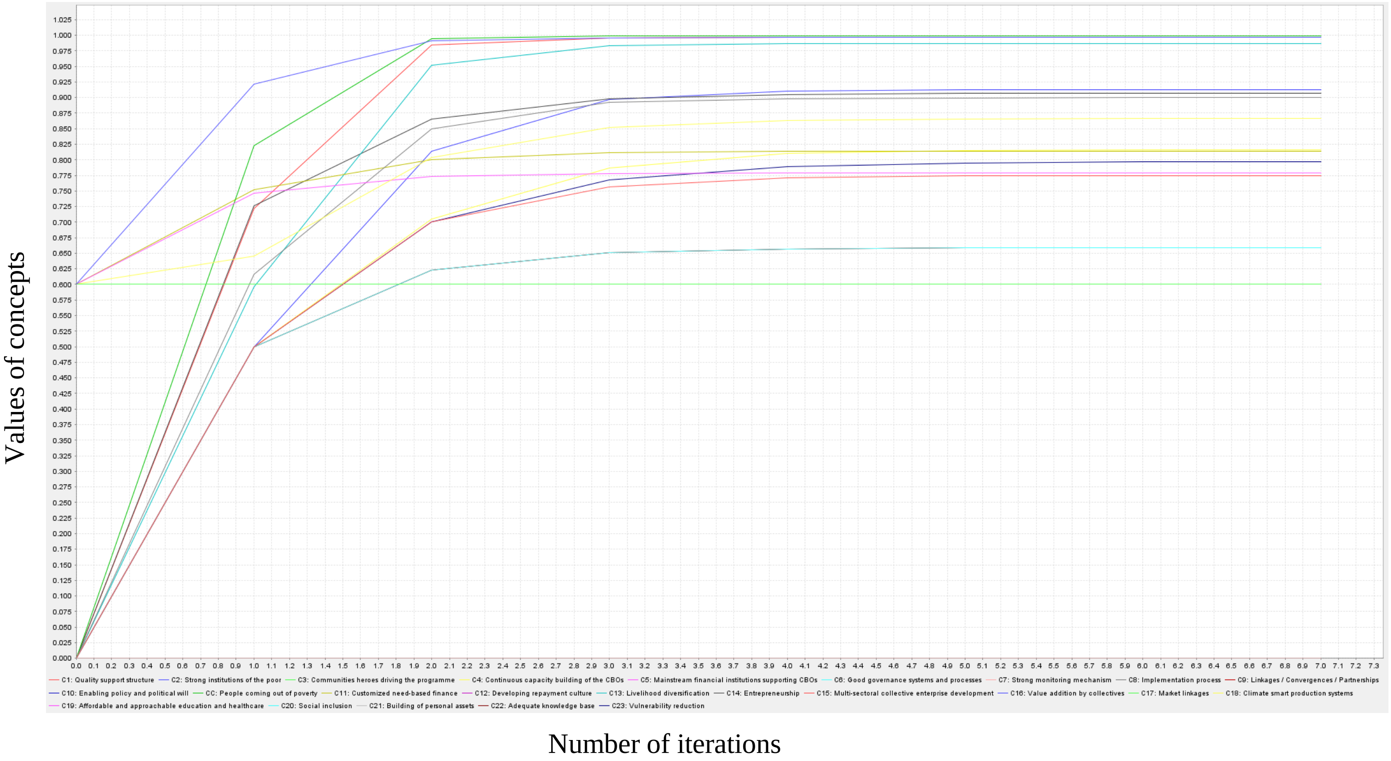

Activation value (0.7): Modified activation rule with sigmoid transformation function

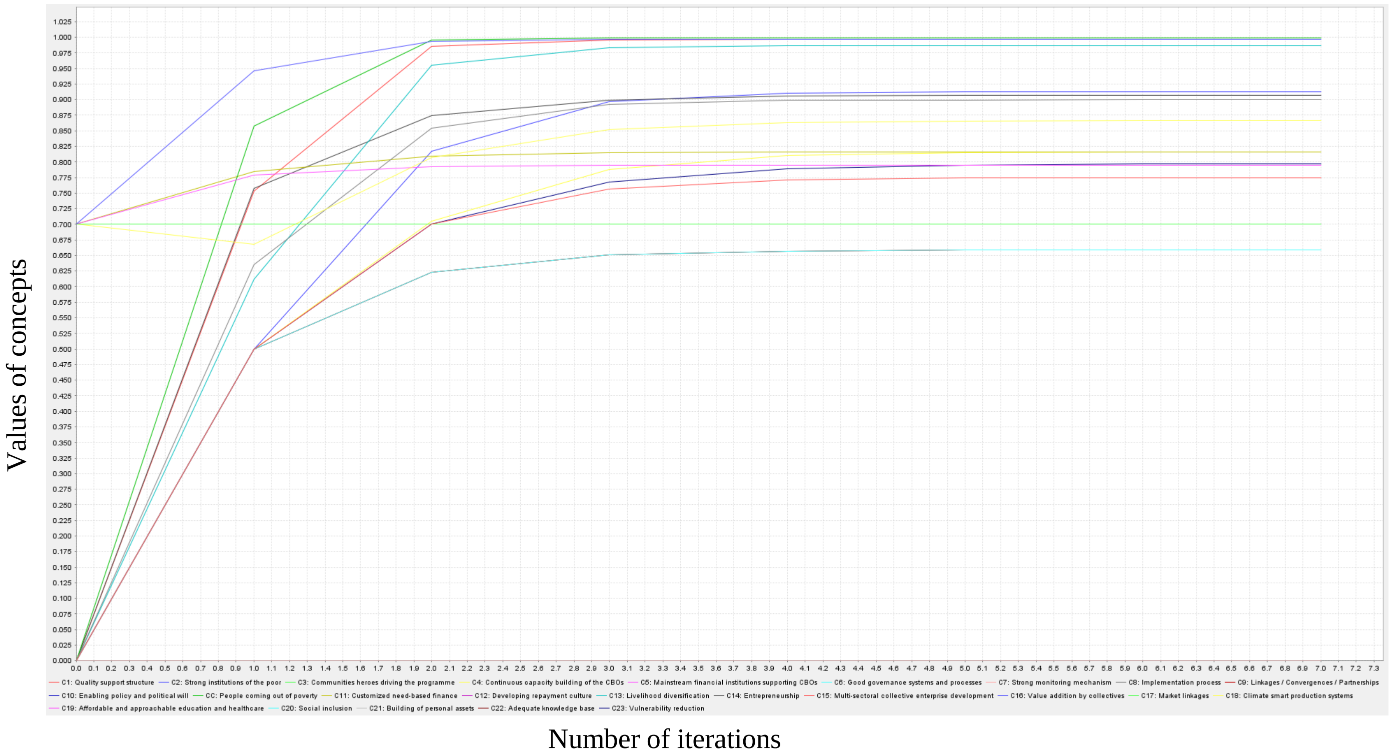

Activation value (0.8): Modified activation rule with sigmoid transformation function

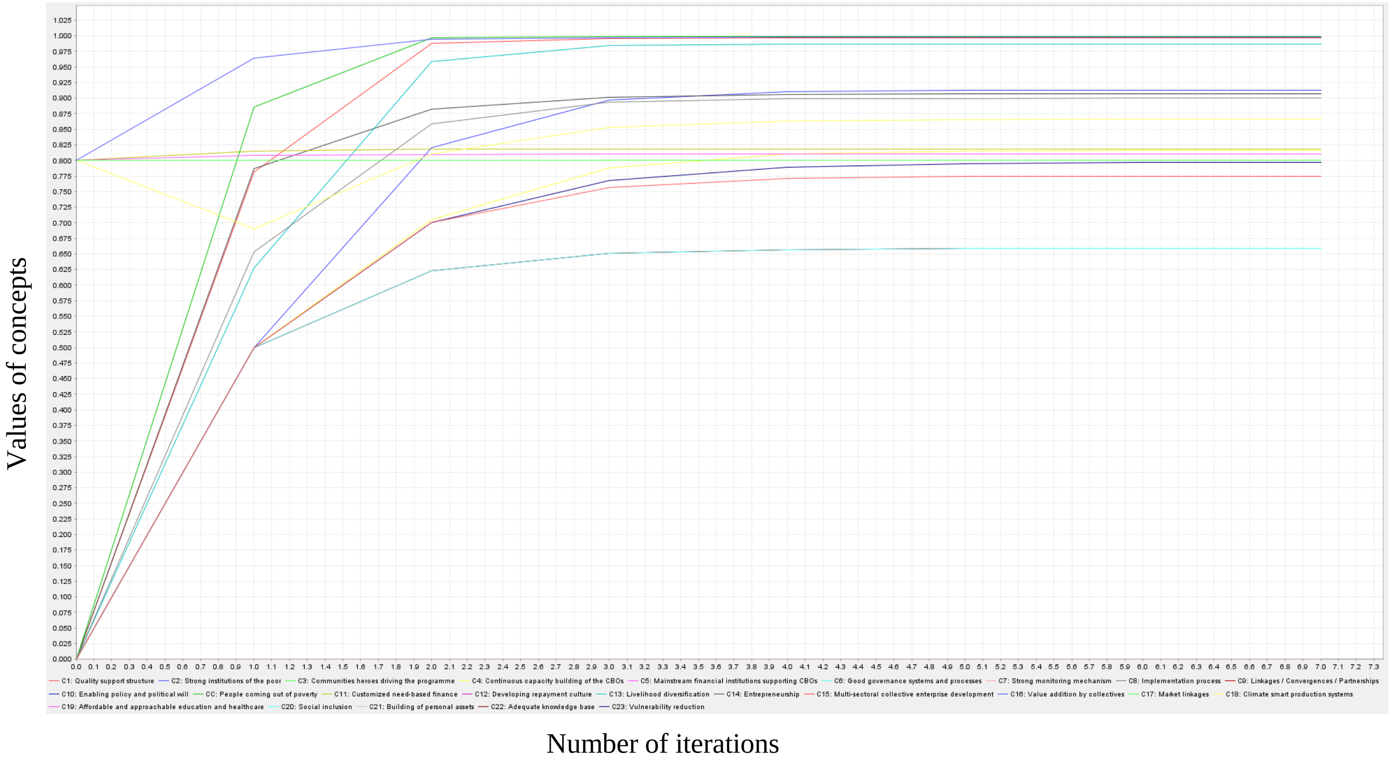

Activation value (0.9): Modified activation rule with sigmoid transformation function

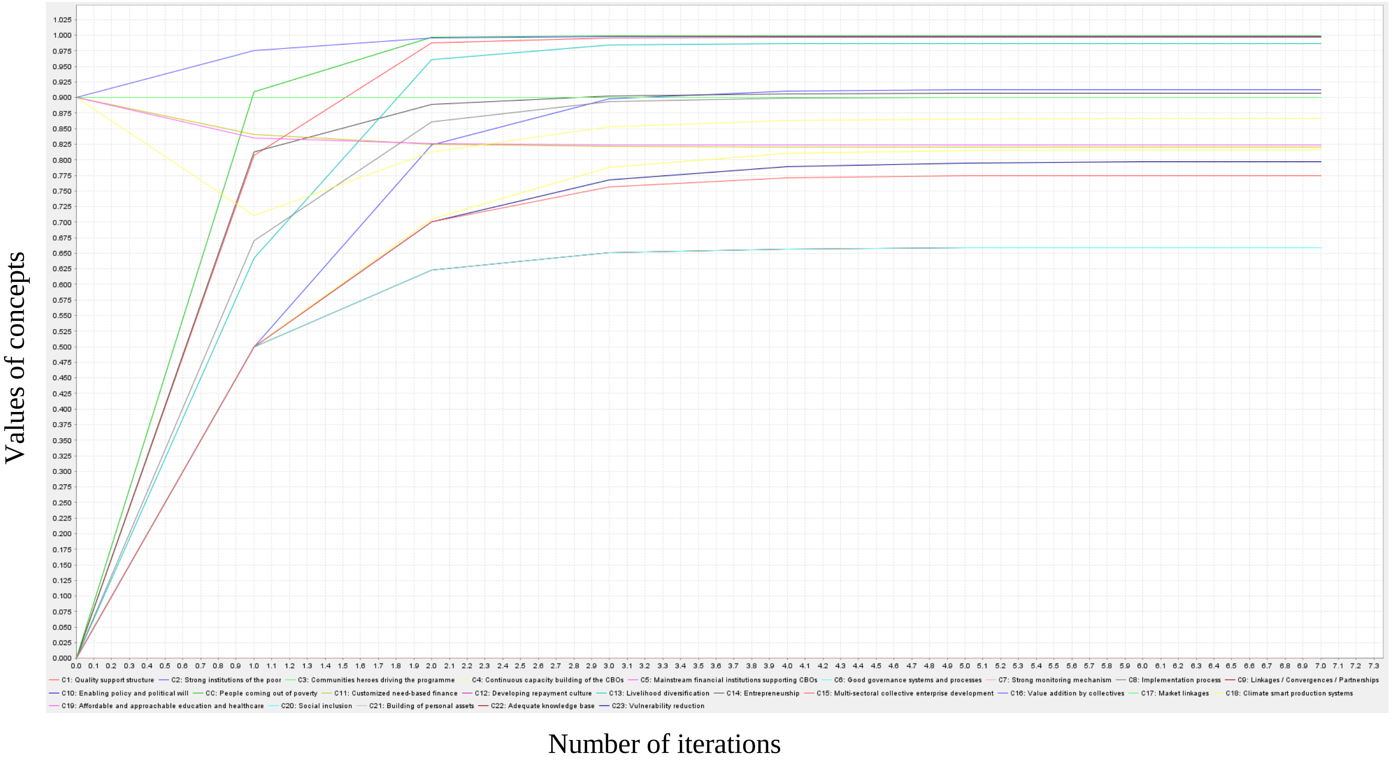

Supplement: S4 Fig — (PDF) [file pone.0227176.s004.pdf]
