# Supplementary material for: Evaluating poverty alleviation strategies in a developing country
Source: PLoS One. 2020 Jan 13;15(1):e0227176. doi: 10.1371/journal.pone.0227176 (PMC6957162; doi:10.1371/journal.pone.0227176)

S5 Fig. Various scenarios for poverty alleviation

Scenario 1: High-quality CBO based micro-finance—Input vector 1: C2, C3, C4, C5, C11, and C12

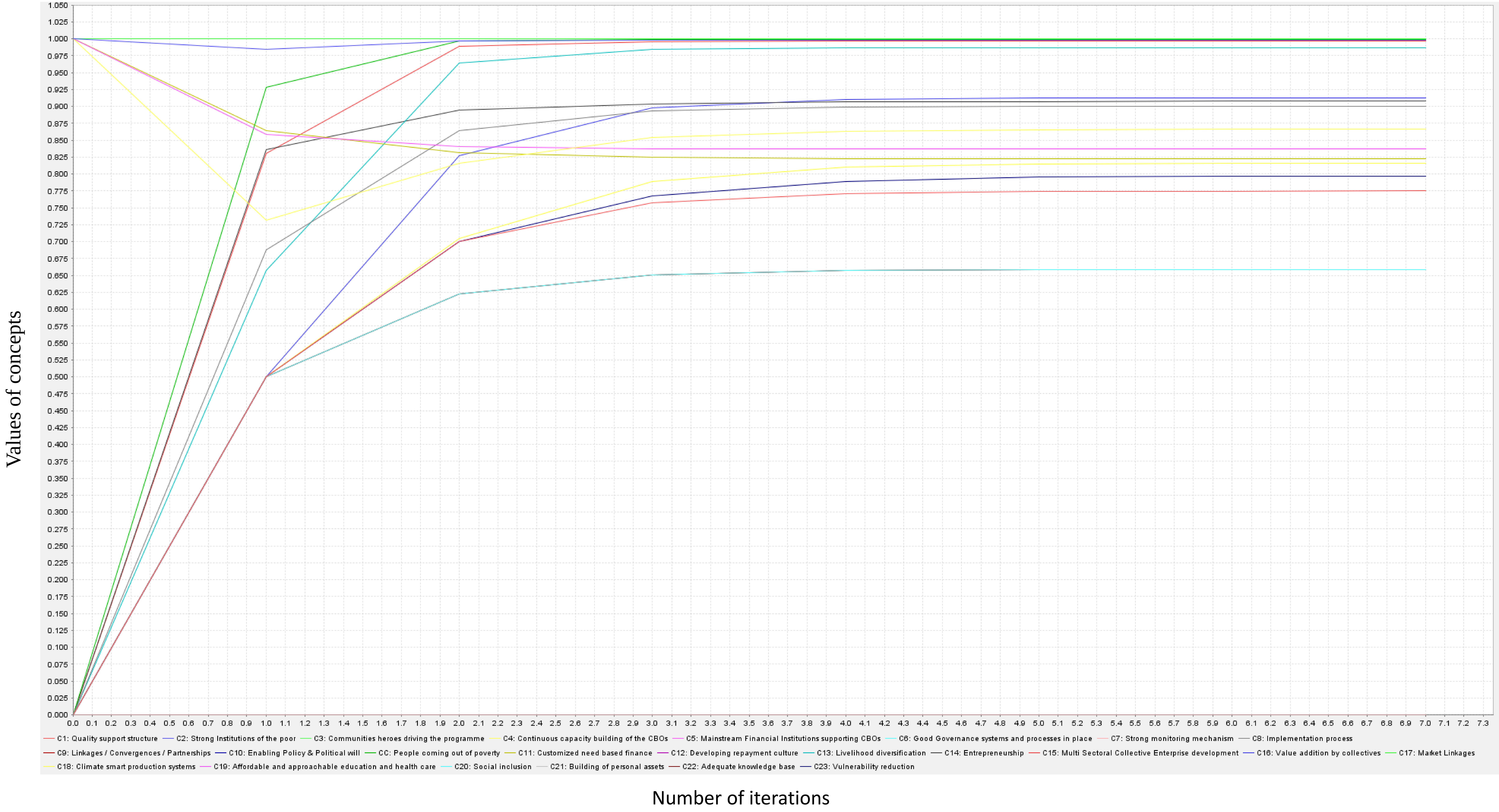

Scenario 2: Capability approach—Input vector 2: C19, C21, and C22

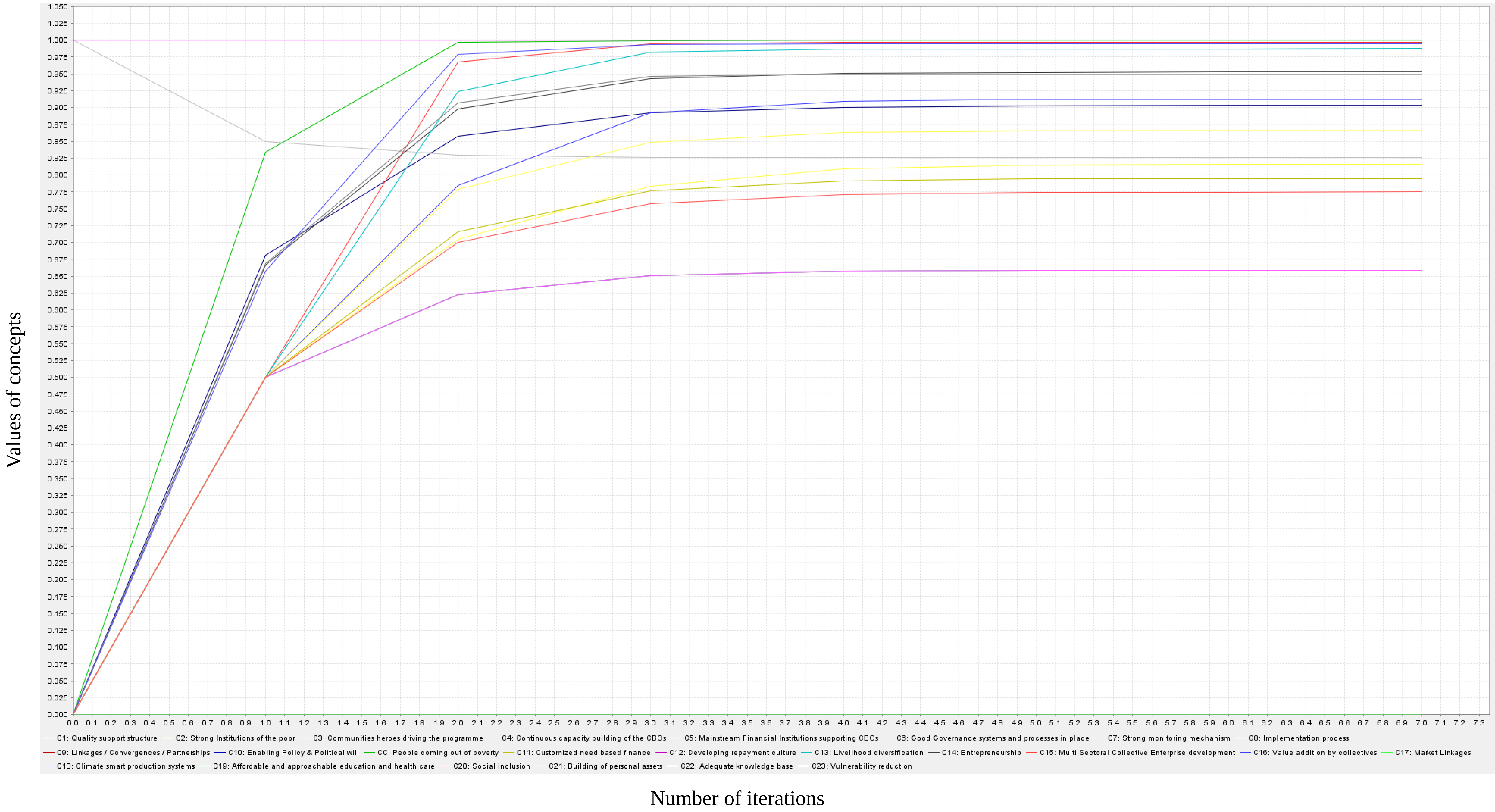

Scenario 3: Market-based approach—Input vector 3: C13, C14, C15, C16, and C17

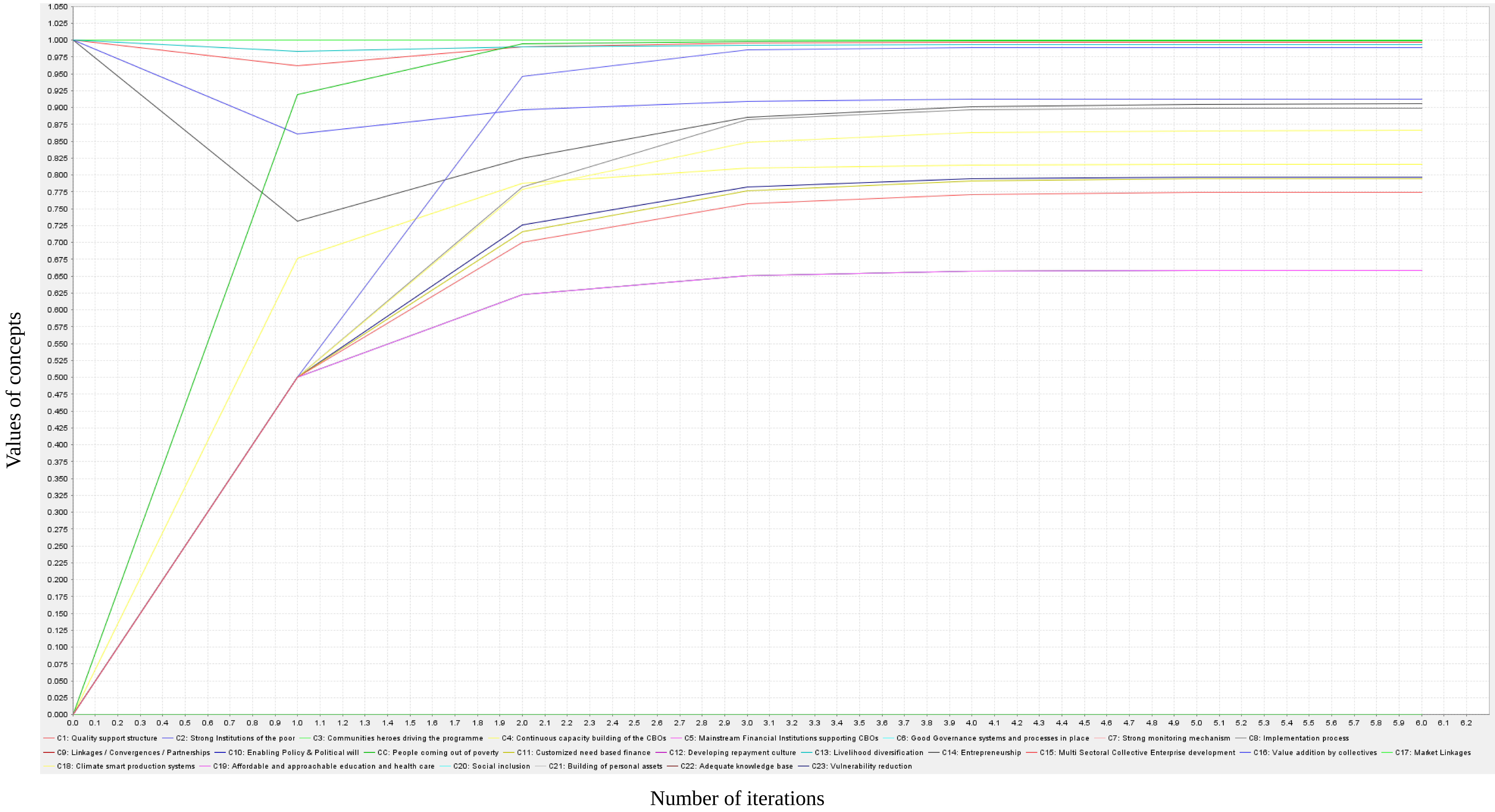

Scenario 4: Capability and social security approach—Input vector 4: C19, C20, C21, C22, and C23

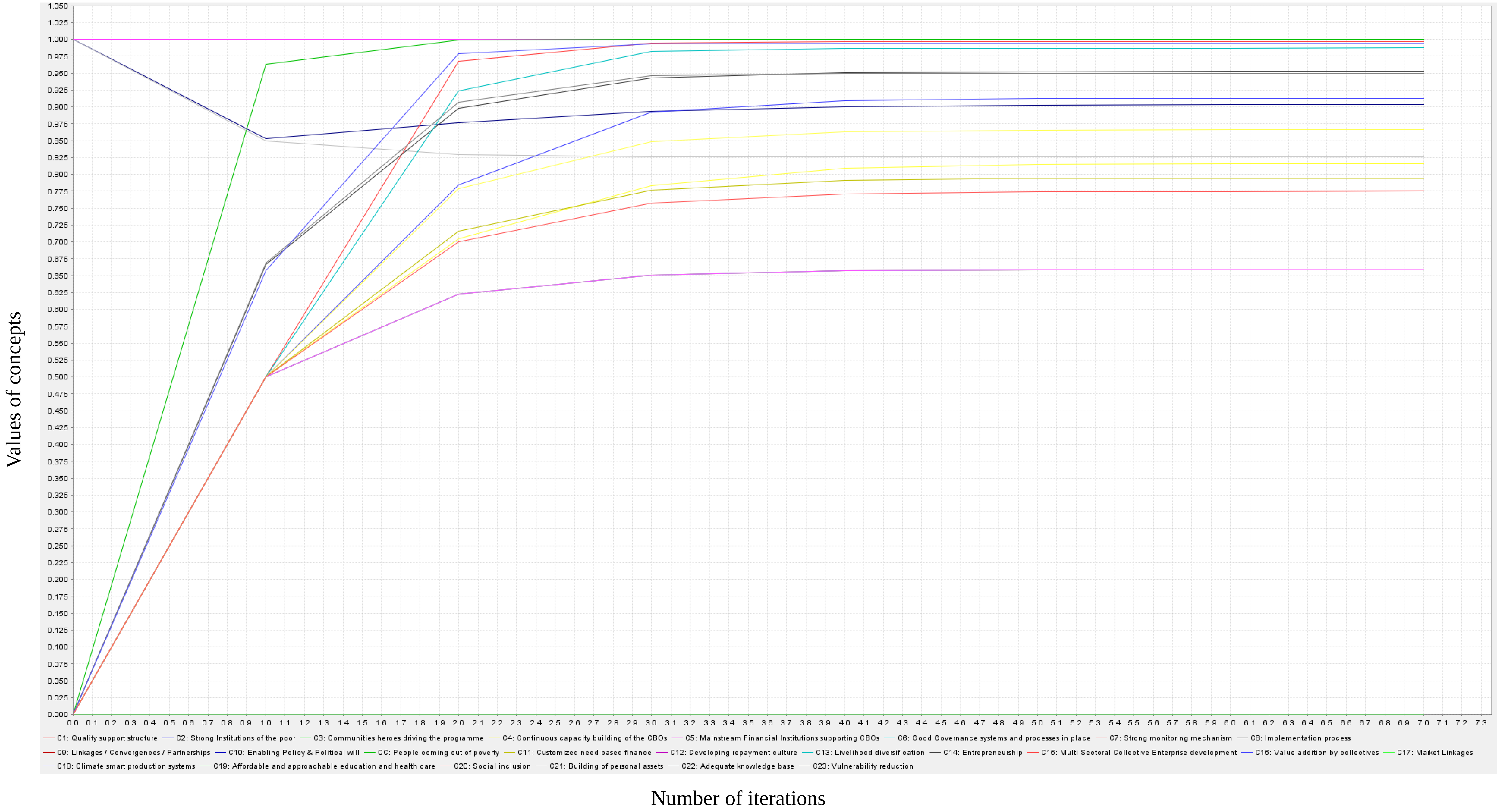

Scenario 5: Good governance—Input vector 5: C1, C6, C7, C8, C9, and C10

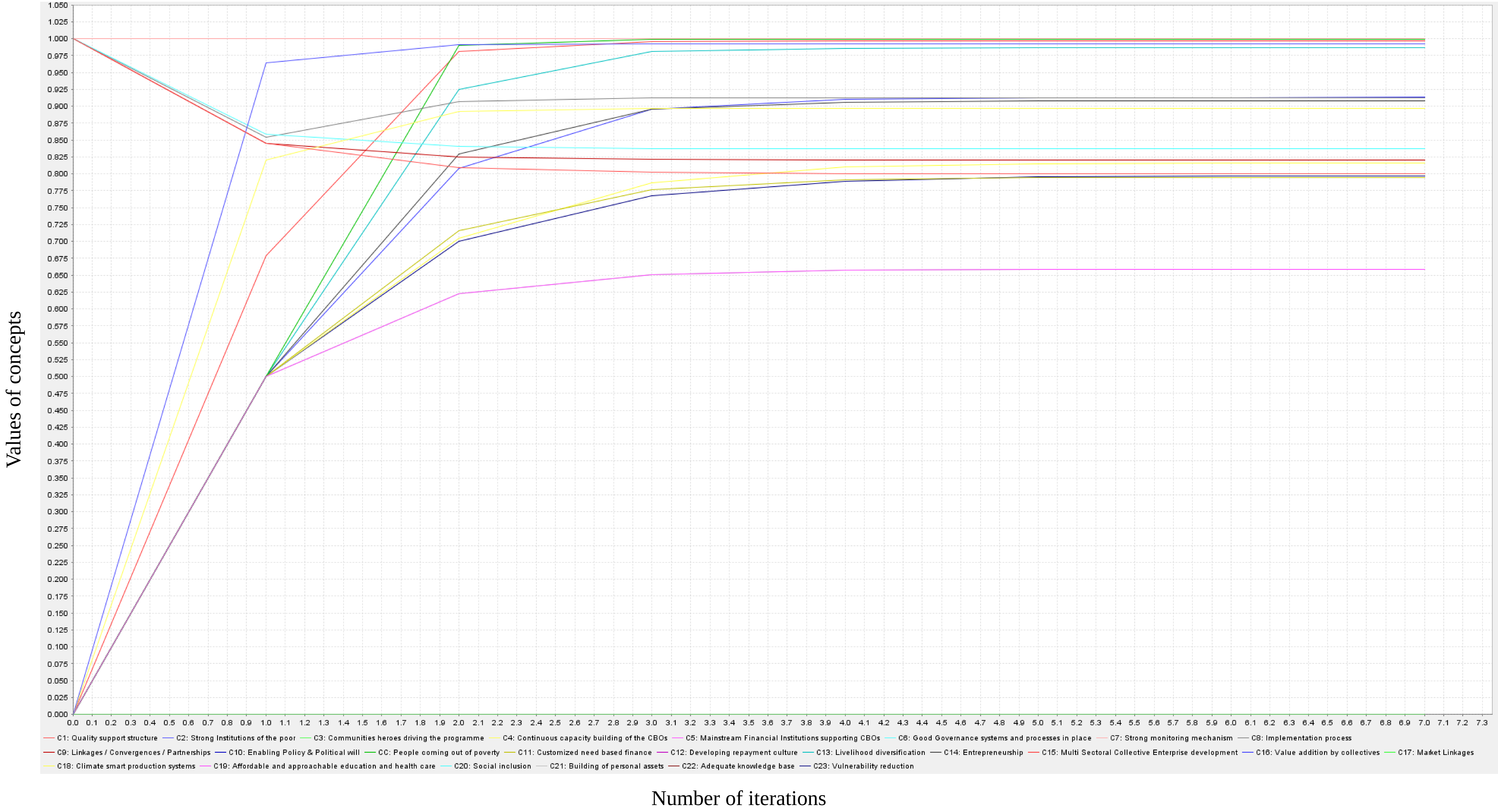

Scenario 6: Integrative approach 1—Input vector 6: C1, C2, C3, C6, C9, C10, C14, and C19

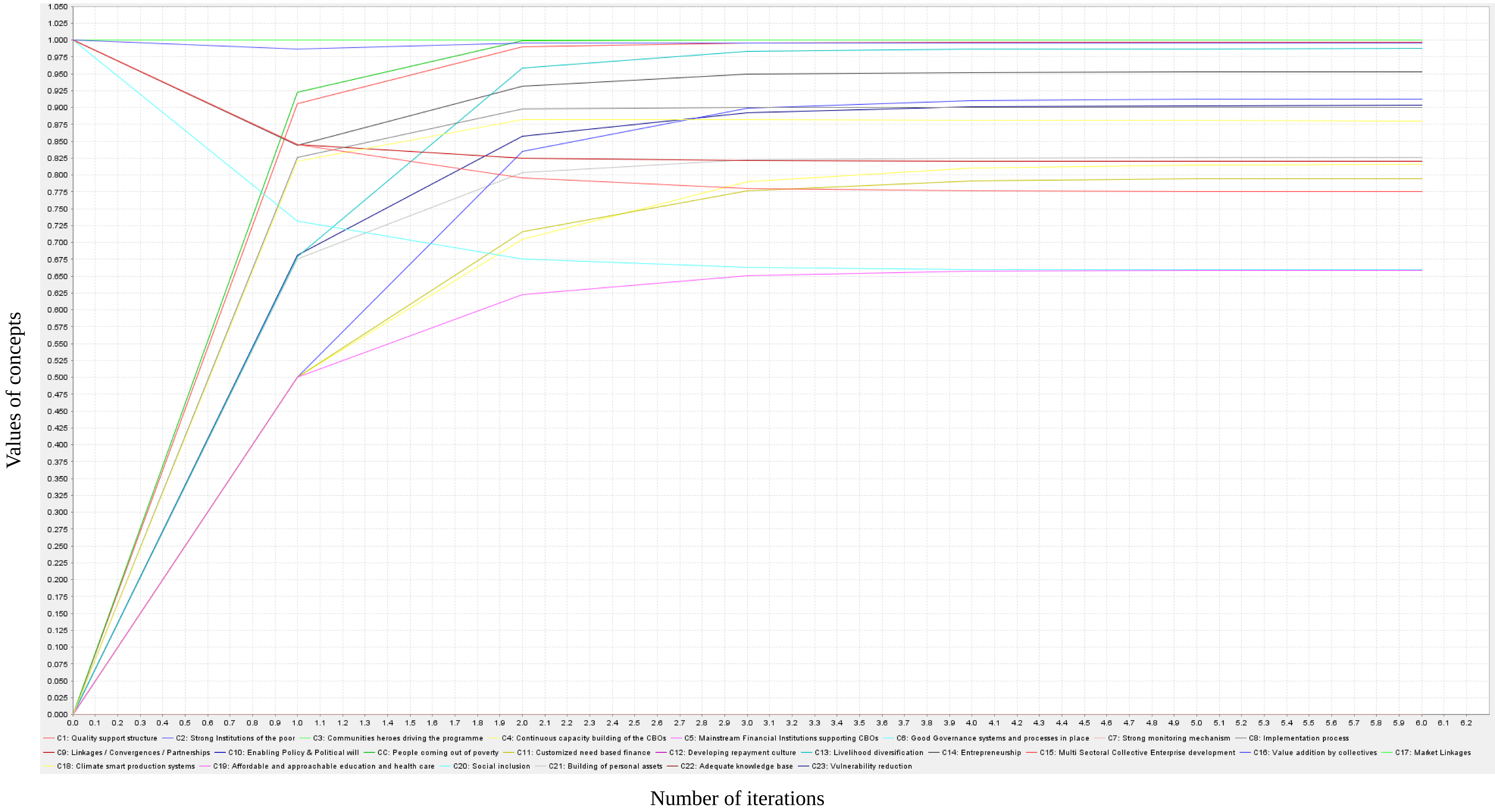

Scenario 7: Integrative approach 2—Input vector 7: C1, C2, C3, C6, C9, C10, C13, and C19

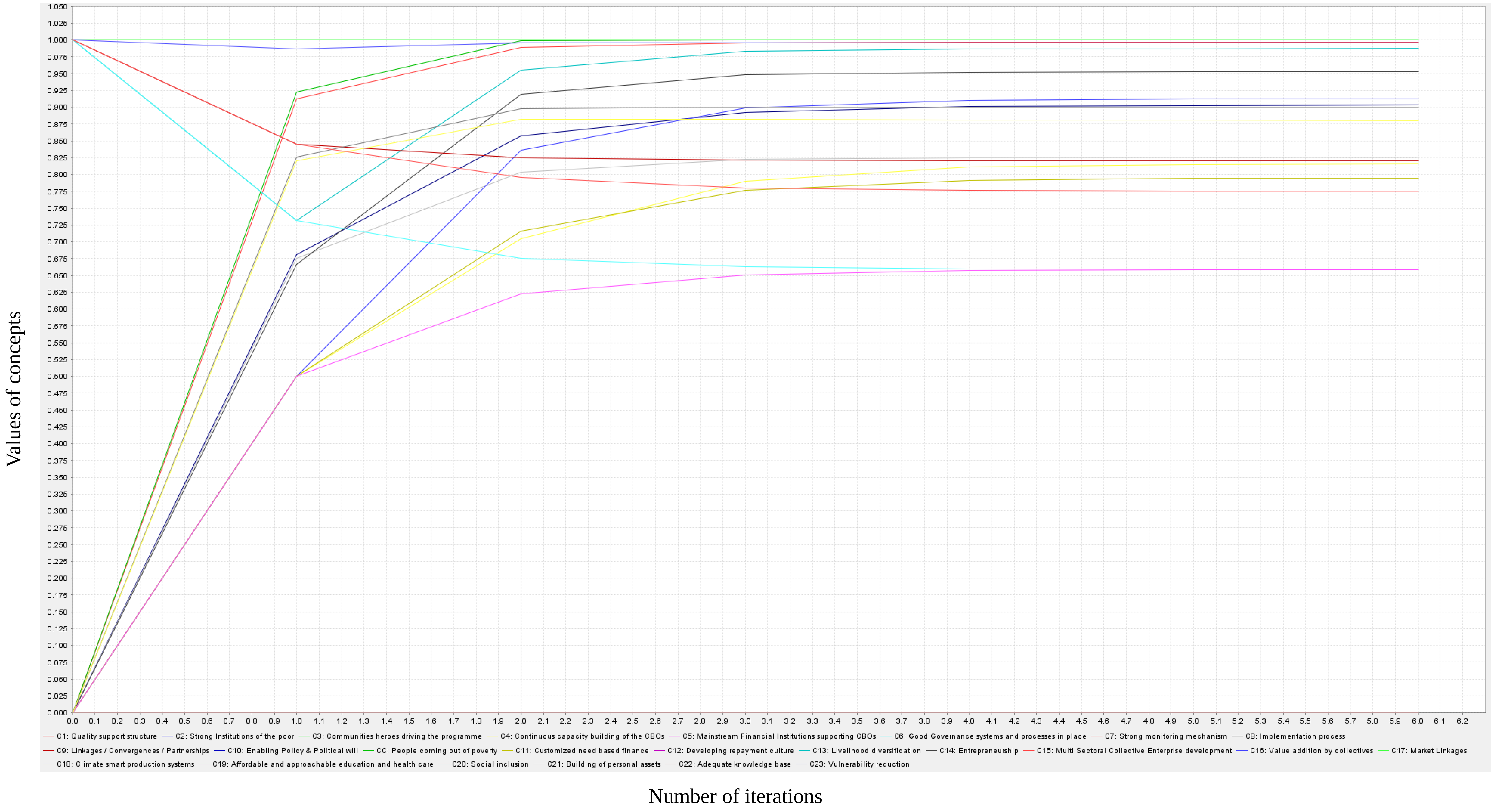

Supplement: S5 Fig — (PDF) [file pone.0227176.s005.pdf]
