# Supplementary material for: Evaluating poverty alleviation strategies in a developing country
Source: PLoS One. 2020 Jan 13;15(1):e0227176. doi: 10.1371/journal.pone.0227176 (PMC6957162; doi:10.1371/journal.pone.0227176)
Supplement: Attachment — Submitted filename: Response to Reviewers.docx [file pone.0227176.s007.docx]

**Authors’ Responses to Comments by Academic Editor**

We thank the Academic Editor and the two anonymous reviewers for providing insightful comments and constructive suggestions.

| **Sr** | **Comments by the Academic Editor** | **Responses of Authors** |
| --- | --- | --- |
| 1 | Thank you for including your ethics statement: "Research Ethics Committee of our Institute approved the methodology of this paper, including the neutral problem domain— direct and consequential impacts of deforestation. Collection of plant, animal, or other materials are not involved in this study"  a) Please amend your current ethics statement to include the full name of the ethics committee/institutional review board(s) that approved your specific study.  b) Once you have amended this/these statement(s) in the Methods section of the manuscript, please add the same text to the “Ethics Statement” field of the submission form (via “Edit Submission”). | Ethics Committee of the Institute of Rural Management Anand known as ‘*Research Ethics Committee*’ approved the instruments as well as the methodology of this research. |
| 2 | In your Data Availability statement, you have not specified where the minimal data set underlying the results described in your manuscript can be found. PLOS defines a study's minimal data set as the underlying data used to reach the conclusions drawn in the manuscript and any additional data required to replicate the reported study findings in their entirety. All PLOS journals require that the minimal data set be made fully available. For more information about our data policy, please see <http://journals.plos.org/plosone/s/data-availability>.  Upon re-submitting your revised manuscript, please upload your study’s minimal underlying data set as either Supporting Information files or to a stable, public repository and include the relevant URLs, DOIs, or accession numbers within your revised cover letter. For a list of acceptable repositories, please see <http://journals.plos.org/plosone/s/data-availability#loc-recommended-repositories>. Any potentially identifying patient information must be fully anonymized.  Important: If there are ethical or legal restrictions to sharing your data publicly, please explain these restrictions in detail. Please see our guidelines for more information on what we consider unacceptable restrictions to publicly sharing data: <http://journals.plos.org/plosone/s/data-availability#loc-unacceptable-data-access-restrictions>. Note that it is not acceptable for the authors to be the sole named individuals responsible for ensuring data access.  We will update your Data Availability statement to reflect the information you provide in your cover letter. | The aggregated condensed matrix (social cognitive map) is given in S1 Table. One can replicate the findings of this study by analyzing this weight matrix. |

**Reviewer's Responses to Questions**

| **Sr** | **Questions** | **Reviewer #1** | **Reviewer #2** | **Authors’ Responses** |
| --- | --- | --- | --- | --- |
| 1 | Is the manuscript technically sound, and do the data support the conclusions? | Yes | Yes |  |
| 2 | Has the statistical analysis been performed appropriately and rigorously? | Yes | Yes |  |
| 3 | Have the authors made all data underlying the findings in their manuscript fully available? | Yes | Yes |  |
| 4 | Is the manuscript presented in an intelligible fashion and written in standard English? | No | Yes | Extensive editing of English language and style is made in this version of the manuscript |

**5. Review Comments to Authors**

**Authors’ Responses to Comments by Reviewers**

**Reviewer #1:**

| **Sr** | **Comments by Reviewer #1** | **Authors’ Responses** |
| --- | --- | --- |
| 5a | This is an interesting research which identifies the critical factors for poverty alleviation in India with the aid of fuzzy cognitive maps (FCMs). This paper has many strengths and some opportunities for improvement, which I will elaborate below: | - We thank you very much for your appreciation and insightful comments, which helped improve the paper. We have tried to address all your concerns. |
| 5b | Section 1 presents interesting information. However, it fails to set out any specific interest to a broader audience.  There is nothing more than a sort of putting forward the topic.  However, what about contribution to relevant literature? Which gaps do you want to fill and how? | **Proposed Contribution of the Paper:**   - Several poverty alleviation programmes around the world affirm that socio-political inclusion of the poor and vulnerable, improvement of social security, and livelihood enhancement coupled with activities including promoting opportunities for socio-economic growth, facilitating gender empowerment, improving facilities for better healthcare and education, and stepping up vulnerability reduction are central to reducing the overall poverty of poor and vulnerable communities (World Bank, 2015; World Bank, 2001). These poverty alleviation programmes remain instruments of choice for policy-makers and development agencies even as they showcase mixed achievements in different countries and localities attributable to various economic and socio-cultural characteristics, among other things. Several poverty alleviation programmes continue to perform poorly despite significant investments (Yalegama, et al., 2012). The failure rate of the World Bank’s development projects was above 50% in Africa until 2000 (Ika and Diallo, 2012). Hence, identifying context-specific factors critical to the success of poverty alleviation programmes is vital. - Rich literature is available pertaining to the conceptual aspects of poverty alleviation. Extant literature emphasises the importance of enhancing capabilities and providing social safety, arranging high-quality community organisation based micro-financing, working on economic development, and ensuring good governance. However, the literature is scanty with regard to comparative performances of the above approaches. The paper tries to fill this gap. This study, through fuzzy cognitive mapping (FCM)-based simulations, evaluates the efficacy of these approaches while calling for an integrative approach involving actions on all dimensions to eradicate the multi-dimensional nature of poverty. Besides, the paper aims to make a two-fold contribution to the FCM literature: i) knowledge capture and sample adequacy, and ii) robustness of the dynamic system model. |
| 5c | Methodology is unclear. Initially a short resume can be proposed to explain several steps.  The methodology used must be linked to the existing literature on FCM.  What is its potential? Its limit? | - Suggested changes have been made in the methodology section for a clear understanding to readers incorporating the new existing literature on FCM. - We adopted the multi-step FCM methodology discussed in the following sub-sections. We adopted the multi-step FCM methodology discussed in the following sub-sections. We obtained individual cognitive maps from the participants in two stages: ‘open-concept design’ approach followed by the ‘pre-concept design’ approach. We coded individual cognitive maps into adjacency matrices and aggregated individual cognitive maps to form a social cognitive map. An FCM-based simulation was used to build policy scenarios for poverty alleviation using different input vectors. - The strengths and applications of FCM methodology, focusing on mental models, vary in terms of approach. It is important to remember, though, that (i) The FCM approach is not driven by data unavailability but is responsible for generating data (Kok, 2009; Özesmi and Özesmi, 2004). (ii) FCMs can model complex and ambiguous systems revealing hidden and important feedback within the systems (Singh and Chudasama, 2017; Falcone, et al., 2017; Kok, 2009; Özesmi and Özesmi, 2004; Kosko, 1986). (iii) FCMs have the ability to represent, integrate, and compare data – an example being expert opinion vis-à-vis indigenous knowledge – from multiple sources while divulging divergent viewpoints (Özesmi and Özesmi, 2004). (iv) Finally, FCMs enable various policy simulations through an interactive scenario analysis (Falcone, et al., 2017; Singh and Chudasama, 2017; Kok, 2009; Özesmi and Özesmi, 2004). - The FCM methodology does have its share of weaknesses. To begin with: (i) Respondents’ misconceptions and biases tend to get encoded in the maps (Singh and Chudasama, 2017; Özesmi and Özesmi, 2004). (ii) Possibility of susceptibility to group power dynamics in a group model-building setting cannot be ruled out. (iii) FCMs require a large amount of post-processing time (Diniz, et al. 2015). (iv) The FCMs-based simulations are non-real value and relative parameter estimates and lack spatial and temporal representation (Özesmi and Özesmi, 2004; Kim and Lee, 1998; Schneider, et al. 1998). - These drawbacks notwithstanding, we, along with many researchers, conceded that the strengths and applications of FCM methodology outweighed the former, particularly with regard to integrating data from multiple stakeholders with different viewpoints. |
| 5d | Results must be linked to the methodology. Please define the relationship and relate your finding with the relevant literature. | - The only explanation of structural analysis was missing in the earlier version of the paper. Suggested changes have been made in the results and discussions to define the relationships as per relevant literature. |
| 5e | Finally, an extensive editing of English language and style is required. | - Editing of English language and style is made in this manuscript as suggested. |

**Reviewer #2:**

| **Sr** | **Comments by Reviewer #2** | **Authors’ Responses** |
| --- | --- | --- |
| 5a | The paper is accurate in the description of the methodology; however, some steps can be explained better. | - We thank you very much for your appreciation and insightful comments, which helped improve the paper. We have tried to explain all the steps of the methodology in sufficient detail. |
| 5b | In 189-190 you explained that the concepts were elicited asking the participants the “critical factors required to ensure that people come out of poverty on a sustainable basis”.  Was this enough to prompt the contribution of the participants or did you give some other information to elicit their contribution.  The quantity of information given before the participant tasks is a question that matter in FCMs building since a great quantity of information could lead to bias while very little information can lead to scanty results.  *How did you reach the correct trade-off?* | - We were very conscious of not to influence the participants’ views during the FCMs building process. - The participants of this research included the designers and implementers of the world’s largest poverty alleviation programme. They had a good understanding of the subject matter and wanted to reflect on the programme. - A major proportion of the literature on fuzzy cognitive maps reflects an ‘open-concept design’ approach, while some studies also rely on a ‘pre-designed concept’ approach with regard to data collection. - In the case of the ‘open-concept design’ approach, concepts are determined entirely by participants and are unrestricted (Singh, et al., 2019; Singh and Chudasama, 2017a; Singh and Chudasama, 2017b; Solana-Gutiérrez, et al., 2017; Diniz, et al., 2015; Gray, et al., 2015; Singh and Nair, 2014; Vanwindekens, et al., 2013; Özesmi and Özesmi, 2004). While the researcher determines the context of the model by specifying the system being modelled, including the boundaries of the system, participants are allowed to decide what concepts will be included. This approach provides a very little restriction in the knowledge capture from participants and can be extremely beneficial especially if there is an insufficient knowledge on the system being modelled. - In the case of the ‘pre-designed concept’ approach, concepts are pre-determined either by experts or by researchers using available literature (Falcone, et al., 2019; Morone, et al., 2019; Falcone, et al., 2018; Ziv, et al., 2018; Falcone, et al., 2017). In this approach, the researcher is able to exercise a higher degree of control over how the system is defined. The ‘pre-designed concept’ approach is likely to be more efficient compared to the ‘open-concept design’ in the context of time required for model building. However, it restricts the diversity of knowledge captured from participants and is able to influence more heavily the way in which this knowledge is contextualised based on input and interpretation. - We have adopted a ‘mixed-concept design’ approach for this study involving data collection in two stages:   **Stage one: ‘Open-concept design’ approach**   - During the first stage, we engaged with the experts and national-level policy-makers who designed the *Deendayal Antyodaya Yojana*-National Rural Livelihoods Mission (DAY-NRLM), a centrally sponsored programme in India. The DAY-NRLM aims at abolishing rural poverty by promoting multiple livelihoods for the rural poor and vulnerable households. The programme is focussed on organising the rural poor and vulnerable communities into self-help groups (SHGs) while equipping them with means of self-employment. - Participants comprising three experts from the World Bank, nine experts from the National Mission Management Unit of the DAY-NRLM, and 25 monitoring and evaluation experts from 25 states of India created 37 FCMs. A sample map of FCMs obtained from these participants is provided in S1 Fig. - We demonstrated the construction of fuzzy cognitive maps with the aid of a map from a neutral problem domain referring to direct and consequential impacts of deforestation, which had been approved by the ‘Research Ethics Committee’ of our Institute. - A group discussion was held with the participants regarding the issues under investigation subsumed under the title “critical factors required to ensure that people come out of poverty on a sustainable basis”. It prompted them to identify major concepts pertaining to the above. These were listed down on a whiteboard by the researchers. Once the participants had understood the process of drawing a fuzzy cognitive map and identified major concepts responsible for poverty alleviation, they were asked to draw a fuzzy cognitive map individually. The participants used the concepts listed on the whiteboard to draw fuzzy cognitive maps. Many participants added new concepts while drawing the maps. They then connected all the concepts through various links based on their personal understanding. The links, represented by arrows in between concepts, show the direction of influence between them.   **Stage two: ‘Pre-designed concept’ approach**   - During the second stage, an instrument depicting 95 concepts under 22 concept categories was prepared based on the FCMs obtained from participants during the first stage (S2 Fig). The instrument also contained links between the 22 concept categories. ‘Research Ethics Committee’ of our Institute approved this instrument as well. We used this instrument during the second stage to obtain FCMs. - We obtained 123 additional FCMs, of which 20 FCMs were obtained from the Chief Executive Officers along with experts from livelihood, enterprise, and community development domains belonging to the National Mission Management Unit in the states of Bihar, Jharkhand, Madhya Pradesh, and Maharashtra. The remaining 103 FCMs were obtained from 103 district project coordinators, who had agreed to participate in the study. Unlike most FCM-based studies, which usually rely upon 30 to 50 participants, this study involved 174 experts and project implementers. Most participants produced FCMs individually and some in pairs. The 174 participants produced 160 FCMs. - The participants were given the instrument and were instructed to assign weights to each concept, wherever applicable, and leave other cells blank. These weights were assigned based on the concepts’ significance regarding poverty alleviation in India. The instrument was designed to allow participants to add new concepts and/or remove existing ones from the instrument based on their understanding and perceptions. Later, the participants were asked to assign weights to all pre-established links between the 22 concept categories. The instrument also allowed participants to draw new linkages between the categories and/or discard the existing relationships based on their understanding and perceptions. After constructing the FCMs each participant made a presentation to the researchers, which was video-recorded. - During the process, participants added 55 new concepts within the pre-classified 22 concept categories. Five new concepts were added under a new category. The final data comprised 23 concept categories and 155 concepts (S3 Fig). |
| 5c | 523-524 “The larger the value of the final state vectors, better the selected policies.”  This means that all the concept give a desired and positive contribution to the poverty alleviation.  Did all respondent give positive concept or did you declined all in a positive way to make them handy? | - The researchers have not declined the concepts in a positive way to make them handy. - The aim of the study was to understand the factors responsible for poverty alleviation, hence, the participants were asked to paint an ideal situation and identify concepts that would contribute to the poverty alleviation. - Participants phrased the factors responsible for poverty alleviation with a positive connotation. Kindly look at wording of concepts. They all have positive connotation. |
| 5d | The paper aims also at giving a methodological contribution.  I suggest some recent paper to enrich this part: | - We have tried referring the papers suggested by you including other recent papers in order to enrich the methodological contribution of this paper. - The paper makes a two-fold contribution to FCM literature: i) knowledge capture and sample adequacy and ii) robustness of the dynamic system model. FCM sampling is often extended if additional maps keep adding new dimensions/ insights. The saturation of FCM sampling is formally measured by tracking the number of new concepts introduced in subsequent exercises and estimating an accumulation curve of concepts. When the point of saturation is reached data collection is stopped. In most studies, the saturation of FCM sampling is reported at 30–32 maps (Singh and Chudasama, 2017a; Singh and Chudasama, 2017b; Jetter and Kok, 2014; Singh and Nair, 2014; Özesmi and Özesmi, 2004). - This study does demonstrate, however, that in the event of a ‘mixed-concept design’ approach when the participants gain access to concepts already identified by other sets of participant groups the latter continue to add new concepts, making the system much more complex and the data richer. Most FCM-based case studies published in scientific journals have taken weights of the causal interactions between the concepts. This study has not only obtained weights of the causal interactions between the concepts but also obtained weights of each concept. Results of the FCM-based simulations, by and large, match with the most critical concepts identified by participants represented by higher relative weights. This demonstrates the robustness of the system. |
| 5e | In 192 why do you mention only two concepts? | - The reference sentence states: “Once concepts related to the issue under investigation were captured, the participants connected these concepts through links showing the direction of influence between the two concepts” — In earlier version of the manuscript. - Here we meant that the participants connected all the concepts through various links according to their personal understanding, where the links, represented with arrows, between the concepts show the direction of influence between them. - The sentence is re-written as follows in the revised version of the manuscript:   Once the participants had understood the process of drawing a fuzzy cognitive map and identified major concepts responsible for poverty alleviation, they were asked to draw a fuzzy cognitive map individually. The participants used the concepts listed on the whiteboard to draw fuzzy cognitive maps. Many participants added new concepts while drawing the maps. They then connected all the concepts through various links based on their personal understanding. The links, represented by arrows in between concepts, show the direction of influence between them. |
| 5f | The diagrams of the various scenarios are hard to read.  I suggest bar diagrams showing differences with the steady state values of each variable under each scenario. | - The given diagrams of the various scenarios in the supporting information illustrate changes in the state vector of each concept across each iteration, showcasing how the FCM convergence took place during the process of simulation. The bar diagram could only show the initial and steady-state values of the concepts for each scenario, which is already shown in Table 2. Therefore, we strongly believe that the current diagrams is the best representation of the changes in the state vector of each concept across each iteration, showcasing how the FCM convergence tooking place during the process of simulation. |
